# Electrically Triggered Drug Delivery from Novel Electrospun Poly(Lactic Acid)/Graphene Oxide/Quercetin Fibrous Scaffolds for Wound Dressing Applications

**DOI:** 10.3390/pharmaceutics13070957

**Published:** 2021-06-25

**Authors:** Alexa-Maria Croitoru, Yasin Karaçelebi, Elif Saatcioglu, Eray Altan, Songul Ulag, Huseyin Kıvanc Aydoğan, Ali Sahin, Ludmila Motelica, Ovidiu Oprea, Bianca-Maria Tihauan, Roxana-Cristina Popescu, Diana Savu, Roxana Trusca, Denisa Ficai, Oguzhan Gunduz, Anton Ficai

**Affiliations:** 1 Department of Science and Engineering of Oxide Materials and Nanomaterials, Faculty of Applied Chemistry and Materials Science, University Politehnica of Bucharest, Gh. Polizu St. 1-7, 060042 Bucharest, Romania; alexa_maria.croitoru@upb.ro (A.-M.C.); ludmila.motelica@upb.ro (L.M.); ovidiu.oprea@upb.ro (O.O.); roxana_doina.trusca@upb.ro (R.T.); denisa.ficai@upb.ro (D.F.); 2 Center for Nanotechnology & Biomaterials Application and Research (NBUAM), Department of Bioengineering, Faculty of Engineering, Marmara University, 34722 Istanbul, Turkey; yasinkaracelebi@marun.edu.tr; 3 Center for Nanotechnology & Biomaterials Application and Research (NBUAM), Department of Metallurgical and Materials Engineering, Faculty of Technology, Marmara University, 34722 Istanbul, Turkey; elifsaatcioglu@marun.edu.tr (E.S.); erai.altan@marmara.edu.tr (E.A.); 4 Center for Nanotechnology & Biomaterials Application and Research (NBUAM), Department of Metallurgical and Materials Engineering, Institute of Pure and Applied Sciences, Marmara University, 34722 Istanbul, Turkey; ulagitu1773@gmail.com; 5 Department of Electrical and Electronics Engineering, Faculty of Engineering, Marmara University, 34722 Istanbul, Turkey; huseyin.kivanc@marun.edu.tr; 6 Genetic and Metabolic Diseases Research and Investigation Center, Department of Biochemistry, Faculty of Medicine, Marmara University, 34722 Istanbul, Turkey; alisahin@marmara.edu.tr; 7 Research and Development Department, The National Institute for Research & Development in Food Bioresources, Dinu Vintila St. 6, 021102 Bucharest, Romania; bianca.tihauan@bioresurse.ro or; 8 Research Institute of the University of Bucharest—ICUB, Spl. Independentei 91-95, 50567 Bucharest, Romania; 9 Research & Development for Advanced Biotechnologies and Medical Devices, SC Sanimed International Impex SRL, 087040 Călugareni, Romania; 10 “Horia Hulubei” National Institute for Research & Development in Physics and Nuclear Engineering, Reactorului, No. 30, 077125 Magurele, Romania; roxana.popescu@nipne.ro (R.-C.P.); dsavu@nipne.ro (D.S.)

**Keywords:** polylactic acid, graphene oxide, quercetin, electrospinning, electrically drug delivery, antimicrobial activity, personalize medicine

## Abstract

The novel controlled and localized delivery of drug molecules to target tissues using an external electric stimulus makes electro-responsive drug delivery systems both feasible and desirable, as well as entailing a reduction in the side effects. Novel micro-scaffold matrices were designed based on poly(lactic acid) (PLA) and graphene oxide (GO) via electrospinning. Quercetin (Q), a natural flavonoid, was loaded into the fiber matrices in order to investigate the potential as a model drug for wound dressing applications. The physico-chemical properties, electrical triggering capacity, antimicrobial assay and biocompatibility were also investigated. The newly fabricated PLA/GO/Q scaffolds showed uniform and smooth surface morphologies, without any beads, and with diameters ranging from 1107 nm (10%PLA/0.1GO/Q) to 1243 nm (10%PLA). The in vitro release tests of Q from the scaffolds showed that Q can be released much faster (up to 8640 times) when an appropriate electric field is applied compared to traditional drug-release approaches. For instance, 10 s of electric stimulation is enough to ensure the full delivery of the loaded Q from the 10%PLA/1%GO/Q microfiber scaffold at both 10 Hz and at 50 Hz. The antimicrobial tests showed the inhibition of bacterial film growth. Certainly, these materials could be loaded with more potent agents for anti-cancer, anti-infection, and anti-osteoporotic therapies. The L929 fibroblast cells cultured on these scaffolds were distributed homogeneously on the scaffolds, and the highest viability value of 82.3% was obtained for the 10%PLA/0.5%GO/Q microfiber scaffold. Moreover, the addition of Q in the PLA/GO matrix stimulated the production of IL-6 at 24 h, which could be linked to an acute inflammatory response in the exposed fibroblast cells, as a potential effect of wound healing. As a general conclusion, these results demonstrate the possibility of developing graphene oxide-based supports for the electrically triggered delivery of biological active agents, with the delivery rate being externally controlled in order to ensure personalized release.

## 1. Introduction

Skin is the largest tissue, and acts as a barrier to temperature, water and pressure, while also protecting the body by not allowing the passage of foreign bodies. Chronic injuries disrupt the integrity of the skin and lead to improper behavior [1]. The use of topical dressings that possess properties necessary to increase the healing process of wounds can play a vital role in wound healing management. Nowadays, modern nano- and microfiber-based dressings can act as a barrier for bacterial infections, maintaining an appropriate humidity, and adsorbing the exudates, thus accelerating the healing process and supporting the reconstruction of damaged tissue by mimicking the architecture of the extracellular matrix (ECM) [2,3]. Biomaterials produced using nano- and microtechnology are often required in the case of large-scale skin loss or in the case of infections [4].

Polymeric nano- and microfibers developed via the electrospinning technique [5] have been demonstrated to be an ideal support for wound healing applications. These fibers have the ability to incorporate various therapeutic substances with bacteriostatic or bactericidal activity, accelerating the healing process of the wounds. PLA is a synthetic biopolymer with excellent biocompatibility and biodegradability properties, and is widely used in regenerative medicine, tissue engineering and drug delivery [6,7]. PLA positively affects drug compatibility and drug-release kinetics by providing a hydrophobic barrier against water loss and the environment [8].

Nevertheless, PLA has some disadvantages, such as poor mechanical behavior and low bioactivity. Thus, in order to overcome these problems, PLA needs to be further functionalized and modified [9,10]. Among the diverse materials used in these composites, the addition of GO can be used to improve the mechanical and physical properties of the polymer matrix. It is well known that the unique physico-chemical properties of GO (high surface area, good biocompatibility, mechanical stiffness, etc.) make it an effective drug delivery system for applications in biomedical areas [11,12]. Nagarajan et al. [13] used organic 2D GO nanosheets as reinforcing agents for gelatin in order to increase the mechanical properties. The results showed that tensile stress was significantly improved (200%) and the gelatin biopolymer fibers was reinforced by 70% by adding GO. Moreover, gelatin/GO was found to be biocompatible when evaluated with human osteosarcoma cells (HOS). In recent years, Q (3,3′,4′,5,7-pentahydroxyfavone), a natural flavonoid substance, has gained much attention due to its beneficial properties to human health. It has been found to exert several biological activities such as antioxidant, antitumoral, antimicrobial and antiviral [14]. In addition, it has been reported to exert antidiabetic effect in vivo [15,16], and has shown enhanced wound healing in treated individuals [17,18]. As a drawback, Q has low water solubility and bioavailability, and is therefore not stable for long periods of time, limiting its practical use [19]. To overcome these problems, different delivery systems have been developed, and thus, the use of Q with an appropriate carrier could improve its properties and minimize its degradation process [20].

In modern medicine, novel controlled and localized drug delivery systems need to be developed in order to allow higher efficiency and controllability, and to reduce the side effects when treating different diseases. Nowadays, the use of various techniques, such as NIR radiation, magnetic and ultrasound radiation, electrical stimulation, pH-controlled mechanisms, etc., is enabling greater control over drug delivery of smart nano- and microstructured polymer systems to the target tissues in comparison to traditional drug-release methods without stimulation, which are unable to adapt to changing therapeutic needs [21]. Smart or “stimulus-responsive” polymers have attracted particular fascination with regard to controlled release [22]. One report in the literature designed stable gelatin electrospun mats (ESM) loaded with chlorhexidine, and investigated their drug release properties when using pH as a stimulus. The ESM were cross-linked with 5% (the optimum concentration) Glutaraldehyde (GTA), demonstrating sustained release at pH 7 and burst release at pH 2. It was also shown that ESM loaded with chlorhexidine have bactericidal activity against *E. coli* and *S. epidermidis* at pH 7–8, as well as biocompatibility using keratinocytes and fibroblasts [23]. A recent study, conducted by Yang et al., investigated the antibacterial activity of a Janus wound dressing composed of polyvinylpyrrolidone (PVP) and ethyl cellulose (EC) polymer matrices loaded with ciprofloxacin (CIP) and silver nanoparticles (AgNPs). In vitro tests showed that more than 80% of the CIP was released in the first 30 s at pH 7, and demonstrated a strong antibacterial effect against *S. aureus* and *E. coli* bacteria [24]. In addition to advantages like high precision and minimal invasiveness for implantable devices, when using electric stimuli, the voltage, current, and duration of the stimulus can also be changed, offering additional control and making the electro-responsive drug delivery systems more feasible and desirable [25]. In this context, the scope of this article was to develop novel PLA/GO fibrous scaffolds containing Q as a biological active substance (a model drug) with triggered delivery capacity, using electrospinning as a processing technique. Moreover, the morphological, mechanical, antimicrobial, and in vitro cellular behaviors of the electrospun scaffolds were investigated in detail in order to prove the potential of the scaffolds as wound dressing materials. The electrically triggered delivery of Q from PLA/GO scaffolds may open new perspectives with respect to regeneration, but also for the treatment of different diseases (if proper drugs are used) in a personalized manner, with the delivery rate being able to be adapted according to the needs of the patient.

## 2. Materials and Methods

### 2.1. Materials

PLA 2003D (CAS: 26100-51-6) was purchased from NatureWorks LLC, Minnetonka, MN, USA. D-isomer of 3.5 wt.%, and has a molecular weight around 120.000 g/mol. GO powder was obtained by Hummers’ modified method [26] (~4.2 nm in thickness, ~10–20 layers) and Q (CAS: 6151-25-3) was purchased from Sigma-Aldrich, Taufkirchen, Germany. Dichloromethane (DCM) (CAS: 75-09-2) was purchased from ISOLAB, Wertheim, Germany. Dimethylformamide, ≥99% (DMF) (CAS: 68-12-2) was purchased from Merck, Darmstadt, Germany. Tween 80 (viscous liquid) (CAS: 905-65-6) was obtained from Sigma-Aldrich, Taufkirchen, Germany.

### 2.2. Preparation of the Solutions

GO was synthesized using the modified Hummer’s method presented in our previous literature reports (Scheme 1 presented in [26,27]). Different solutions were prepared at discrete concentrations, as shown in Table 1. PLA granules were dissolved in 20 mL DCM/DMF (1:9) for 2 h to obtain a solution consisting of 10% PLA using the magnetic stirrer (Wise Stir^®^, MSH-20 A, Wertheim, Germany). After mixing, 3% Tween 80 was added into the PLA solution and the solution was stirred for another 15 min. Following that, different concentrations of GO (0.1, 0.5, and 1%) were dispersed in 10 wt.% PLA solution and this mixture was stirred for 1 h. To load the Q, 20 mg of Q was added in each suspension of 10% PLA/(0.1, 0.5, 1%) GO (wt./wt.) and stirred for 1 h.

To determine the physical characterizations of the prepared electrospinning mixtures; density, electrical conductivity, surface tension, and viscosity values were measured before the electrospinning process. Density measurements were carried out using a standard 10 mL bottle. The electrical conductivity was measured using the Cond 3110 SET 1, WTW, Weilheim, Germany. The surface tension values were determined using a force tensiometer Sigma 703D, Attention, Darmstadt, Germany. The viscosity values of the solutions were determined using a DV-E, Brookfield AMETEK, Chandler, AZ, USA instrument.

### 2.3. Electrospinning Process

**Figure 1:**
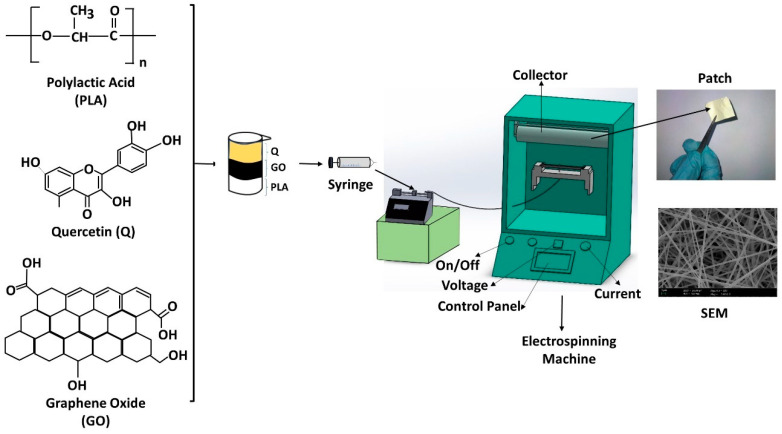
Schematic illustration of the idea, fabrication, and methods.

The experimental setup consisted of a syringe pump (NE-300, New Era Pump Systems, Inc., Farmingdale, NY, USA), a single brass needle (diameter of 1.63 mm), a high-voltage power supply connected to the needle, and a laboratory-scale electrospinning unit (NS24, Inovenso Co., Istanbul, Turkey). The collector can have different shapes and features as per request. An aluminum cylinder covered with grease-proof paper was used to collect the fibers. Then, plastic syringes filled with 20 mL suspensions were prepared. The collector and the tip of the needle were connected to a high-voltage power supply. The electrospinning parameters were optimized during the electrospinning process and found as 25–26.6 kV working voltage range, the distance between the needle and collector was 12 cm, and the flow rate values were ranged between 0.3 and 0.6 mL/h (the schematic illustration of the idea is shown in Figure 1).

### 2.4. Morphological Characterization of the Microfiber Scaffolds

The surface morphological characterizations of the reference and Q-loaded scaffolds were performed by scanning electron microscopy (SEM, EVO LS 10, ZEISS, München, Germany). The average diameters of the scaffolds were determined by measuring 100 fiber diameters using image analysis software (Olympus AnalySIS, Waltham, MA, USA).

### 2.5. Differential Scanning Calorimetry (DSC)

Thermal properties of the fiber scaffolds were evaluated using a differential scanning calorimeter (DSC, Shimadzu, Tokyo, Japan). The temperature range was adjusted from 25 °C to 400 °C at a rate of 25 °C/min.

### 2.6. Mechanical Testing

The Shimadzu (EZ-X, Tokyo, Japan) tensile testing device was used to measure the tensile strength and elongation at break values of the microfiber scaffolds. Firstly, the scaffolds were cut to be 10 mm in width and 50 mm in length before the tensile testing, and three samples were used from each scaffold in order to obtain mean tensile strength and strain values. The thickness values of the scaffolds were measured using a digital micrometer.

### 2.7. Swelling and Degradation Behaviour of the Scaffolds

To evaluate the swelling and degradation behavior, the scaffolds were kept in 10 mL phosphate-buffered saline solution (PBS; pH 7.4) at 37 °C in a thermal shaker. The initial weight of the samples was measured on the first day. From time to time (24, 49, 74 and 93 h) the samples were removed from the PBS solution, surface-dried by patting them with a filter paper, and weighed for the swelling test. The swelling ratio was defined by using the following Equation (1):

(1) $$\mathrm{Swelling}\;\mathrm{ratio}=\frac{W_{f}-W_{i}}{W_{i}}\times 100$$

where: W_f_ is the weight of the wet samples and W_i_ is the initial weight of the dried samples [28].

These experiments also revealed the degradation processes of the samples [29].

### 2.8. Water Vapor Permeation (WVP)

The WVP was determined as described in [30] by using permeation cups of 20 mm diameter sealed with a sample film. Each cup contained 1 g of dried calcium chloride (CaCl_2_). The permeation cups were put in a box at a temperature of 25 °C and 80% relative humidity. Their weight was measured at given intervals for ten days.

### 2.9. In Vitro Drug Release Test

The in vitro release properties of Q from PLA/GO/Q microfibers were determined at pH 7.4 in phosphate buffered saline (PBS) at 37 °C. The resulting Q concentration was determined at different time intervals using an UV-Vis spectrophotometer (Shimadzu, Tokyo, Japan). The linear calibration curve of the drug was determined at a wavelength range of 300–400 nm and for four different (2, 4, 6, 8 μg/mL) drug concentrations. The first step in the drug release test was that 5 mg drug-loaded microfibers were weighed and inserted into Eppendorf tubes with 1 mL PBS (pH 7.4). The absorbance measurements were performed at 15 min, 30 min, 1, 2, 3, and 4 h. At these time points, fresh PBS was used after each measurement. The release profile of the Q was detected at 376 nm.

### 2.10. Q Encapsulation Efficiency

The encapsulation efficiency is the ratio between the mass of existent drug encapsulated and the mass of theoretical drug loaded into the scaffolds. In this study, a standard assay protocol was used to point out the Q content inside the scaffolds. Briefly, the scaffolds were fully dissolved in the solvent and drug detection was carried out using UV absorbance at 376 nm. Q-loaded scaffolds were weighed (nearly 5 mg) and dissolved in 10 mL of solvent in a beaker. After 1 h of mixing, 1 mL of the resulting solution was taken and used to determine the encapsulation efficiency performance of the scaffolds by UV absorbance meaurements. All determinations were performed in triplicate for all scaffolds. The encapsulation efficiency (%) was calculated using the following Equation (2) [28]:

(2) $$\mathrm{Encapsulation}\;\mathrm{efficiency}=\frac{\mathrm{mass}\;\mathrm{of}\;\mathrm{existent}\;\mathrm{drug}\;\mathrm{loaded}\;\mathrm{in}\;\mathrm{patches}}{\mathrm{mass}\;\mathrm{of}\;\mathrm{used}\;\mathrm{in}\;\mathrm{fabricated}\;\mathrm{patches}}\times 100$$

### 2.11. Electrically Controlled Q Release

#### 2.11.1. Design and Setup of the Circuit

In this study, an OTA-CFA-based changeable frequency pulse generator was used to examine the effect of the electric current on drug release at different frequency values. To be more specific, the circuit produced pulse train signals. The circuit consists of three parts. The first part of the circuit is the square waveform generator, whose topology is part of the prototype. A square waveform generator is widely used in medical circuits and biomedical applications, playing a crucial role in these applications [31,32,33]. The circuit consists of an OTA (operational transconductional amplifier) and a CFA (current-feedback amplifier). Saturation levels occurs as high (H) and low (L) because of using OTA in circuit. The potential of node voltage was calculated as (3):

(3) $$k=\frac{R_{1}}{R_{1}+R_{2}}$$

Time period of circuit depends on value of *k* and time constant value of the *RC* (Equation (4)).

(4) T=4kRC

where *T* is time constant (seconds), *C* is the capacitor and *R* is the resistor.

Thus, the square wave was formed at the output of the OTA. The circuit was designed around CFA-OTA [34]. An AD844 was chosen as a commercial CFA. Additionally, an LM13700 was used as the OTA with a biasing current equal to 50 uA. The supply voltage was set to +/− 9 V. *R* = 10 k, *C* = 150 nF, and *k* = 1/2 (*R*_1_ = 10 k, *R*_2_ = 10 k) were chosen. The output of the generator was nearly measured as being +/− 9 V at a frequency of 333 Hz. In the clamping stage, the negative peak of the signal was raised above the zero level, and then the signal was positively clamped. In the positive clamper, a diode, a resistor and a capacitor were used to shift the output signal to the positive portion of the input signal. Another stage of the circuit is the ultra-fast switching stage, in which pulse trains are changed into positive pulse trains and negative pulse trains. An SFH617A optocoupler was applied in the circuit for fast switching and high reliability in isolation. Furthermore, pure positive and negative pulse trains were obtained in the fast switching stage.

The final stage of the circuit is the multiplexer stage. A multiplexer is an electronic device that has multiple inputs and one output. Specifically, the inputs are steered to the output by applying control signals.

The ADG408 was used in this process because it provides a high switching speed and a low resistance. Each channel of the ADG408 conducts equally in both directions. Additionally, in the off condition, the supplies are blocked. A 1-bit binary address line A0 was used to obtain the output from two differential inputs. The binary address was controlled using a function generator as a 0–5 V logic signal. To clearly see the frequency change of the output signal, the TTL (transistor-transistor logic) signal was given to the control pin at different frequencies (10 Hz, 50 Hz, 100 Hz) as 25%, 50%, 75% duty cycle. Therefore, the pulse train output was acquired using the general process.

The simulation was performed in Multisim before constructing the prototype, in order to depict an ideal circuit. Additionally, the duty-cycle was chosen as 50% for the simulation. The output of the general circuit is shown below. The circuit produces +/− 5 V PWM. Moreover, there are pulses within each of the positive and negative trains.

The comprehensive circuit diagram of the electrical stimulation device is provided in the Supplementary Material Figure S1. The circuit diagram was constructed with using Altium Designer.

#### 2.11.2. Electrically Controlled Q Release from the Microfiber Scaffolds

After the electrically controlled release system was set up, approximately 5 mg of all scaffolds loaded with Q were weighed and put into Eppendorf tubes with 1 mL of PBS (pH 7.4). Electric current was transferred to the PBS in the Eppendorf tube with an Ag/Pt electrode. Experiments were performed at frequency values of 10 and 50 Hz under constant voltage (±10 V), and the structures were exposed to electricity for 5, 10, 20, 40, 60, 80, 100 and 120 s. After applying the electric field, the PBS in the Eppendorf tubes was taken, and the absorbance values were determined by UV absorbance measurements at a wavelength of 376 nm.

### 2.12. Antimicrobial Activity Assessment

Evaluation of the antimicrobial activity of the samples was performed by determining the logarithmic and percentage reduction, as well as the recovery rate of microorganisms.

The evaluation was performed using three reference strains from the American Type Culture Collection (ATCC, Manassas, VA, USA): *Staphylococcus aureus* ATCC 6538, *Escherichia coli* ATCC 8739, and *Candida albicans* ATCC 10231. The microbial suspensions of 1.5 × 10^8^ CFU/mL, obtained from fresh 15 to 18 h cultures, developed on solid medium, were adjusted using the nephelometric 0.5 (bacteria) and 1 (fungi) McFarland standard, and then serially diluted to 10^−5^. Samples were cut at 1 cm^2^, and the inoculum volume was adjusted according to the sample surface. The samples were placed in contact with the microbial inoculum for 30 min, thoroughly spun on a vortex, and subsequently, five decimal serial dilutions were carried out in order to determine the logarithmic and percentage reduction of the microbial populations. A total of 10 µL was inoculated in triplicate in spot on Muller Hinton solid medium, or Sabouraud for microfungi. After 18–24 h of incubation at 36 ± 2 °C the plates were read by counting the colonies.

The logarithmic reduction was calculated using the Equation (5):

(5) $$L\;(Logarithmic\;reduction)=l_{g}\;\frac{A}{B}$$

where *A* = no. of viable organisms before treatment; *B* = no. of viable organisms after treatment.

The percentage reduction in microbial populations was calculated using the Equation (6):

*P* = (1 − 10^−*L*^) × 100 (6)

where *P* is the percentage reduction and *L* is the logarithmic reduction.

The determination of the recovery rate was carried out by performing 12 decimal serial dilutions from the inoculated samples with 10^−5^, after 18 to 24 h of incubation. The recovery factor was calculated using the Equation (7):

(7) RF=CFU positive control/ CFU sample

The number of colonies obtained for the sample was compared with the ones obtained on the control plates. The number of colonies counted should not differ by more than a factor of 2 (recovery rate 50–200%).

### 2.13. Biocompatibility Test

To assess the biological effect of the PLA-GO-Quercetin, the L929 fibroblast cell line (ATCC, Manassas, VA, USA) was used. The cells were cultured in Earle’s minimum essential medium (MEM) containing l-glutamine (Biochrom, Merck Millipore, Darmstadt, Germany) and supplemented with 10% fetal bovine serum (FBS) and 1% penicillin and streptomycin antibiotics, in standard conditions of temperature and humidity (37 ± 2 °C, 5 ± 1% CO_2_ and more than 90% humidity). The electrospun thin films were sterilized by UV exposure.

A circular mold of 5 mm diameter and 0.2 mm thickness was used to prepare the samples for the cell test. Sterilized microfiber structures were placed in 96-well plates, and fibroblast cells (L929) were plated onto each of the microfiber structures and incubated in standard conditions of temperature and humidity up to one week. All biological determinations were performed three times.

The cellular viability and proliferation was quantitatively measured using the MTT tetrazolium-salt assay (Serva). For this, at the corresponding time-point, the medium was removed and gently replaced with fresh culture medium containing 10% MTT solution (5 mg/mL in PBS). The cells were incubated for another 3h in standard conditions and afterwards the supernatant was replaced with DMSO, in order to solubilize the grown formazan crystals. The absorbance corresponding to each sample was measured at a wavelength of 540 nm using a microplate reader.

For the fluorescence microscopy morphological investigations, 100,000 L929 cells in 400 µL culture medium were seeded onto each corresponding PLA-GO-Quercetin sample and allowed to attach for 24 h. After this time, the samples were fixed during 10 min using 4% paraformaldehyde in PBS and stained during 10 min using 2 µg/mL Acridine Orange in PBS. Images were acquired using an Olympus BX-51 microscope equipped with an Andor DSD2 Confocal Unit.

An amount of 100,000 cells in 400 µL culture medium were seeded onto each corresponding PLA-GO-Quercetin sample. At the end of the incubation period of time (96 h), the samples were prepared for scanning electron microscopy as follows: after gentle PBS washing, the samples were fixed using 2.5% glutaraldehyde in PBS, during 1h, at room temperature. After another PBS washing, the samples were dehydrated by successive immersion in ethanol and ethanol-hexamethyldisilazane solutions for 15 and 3 min, respectively. At the end of the dehydration processing, the samples were allowed to dry prior to SEM analysis. A thin layer of Au was used to cover the samples, and the image acquisition was performed with an FEI SEM (Hillsboro, OR, USA) with a secondary electron beam of 30 keV energy.

IL-6 cytokine release was measured quantitatively using the ELISA technique (Quantikine ELISA, R&D Systems) at 24 h after L929 cells interaction with 1:8 diluted biomaterial extracts. The extracts were prepared according to the ISO 10993-12:2002(E) standard (Biological evaluation of the medical devices. Part 12: Sample preparation and reference materials). Thus, the extraction ratio (surface area of the sample/cells culture medium volume) was set to 3 cm^2^/mL. The respective samples were placed in culture medium inside closed glass jars, in sterile conditions, in order to prevent contamination. The extraction was done using an orbital shaker for 24 h, at 37 ± 2 °C.

A total of 5000 cells/well were seeded in a 96-well plate and allowed to attach for 24 h in standard conditions. After this period of time, 100 µL of the dilluted freshly obtained sample extract was added into each corresponding well. The measurements were carried out at 24 h. IL-6 was measured in cell culture supernatant samples according to the producer’s specifications. The optical density of each sample was assessed at 450 nm, with a correction at 570 nm and calculated using an IL-6 cytokine standard curve.

### 2.14. Statistical Analysis

Microfiber diameter measurements were performed using the SPSS 17.0 (IBM, Armonk, NY, USA) analysis program. The level of significance was taken as *p* < 0.05 and the data were labeled with (*) for *p* < 0.05, (**) for *p* < 0.01, and (***) for *p* < 0.001. Data are presented as mean ± SD.

## 3. Results and Discussion

The resulting GO is water dispersible (the suspension was ultrasonicated at room temperature for 10 min); after 1 day the suspension is still maintained, with only a slight increase in precipitation (~1 g/L). Zeta potential is around −37.36 mV, which shows a good stability of the suspension. According to Raman spectroscopy, presented in our previous literature reports, the ratio I2D/IG is less than 2, indicating that GO material is in the form of multilayers. Additionally, the individual sheets of GO were found to be less than 20 μm, ~4.2 nm in thickness, and the number of layers was estimated to be between 10 and 20 layers. The Boehm method showed that the total number of functional groups was 1.17 meq/g, with the content of phenolic groups being higher, as they play a very important role in the structure of GO [26].

### 3.1. Physical Characterizations of the Microfiber Suspensions

The physical properties of the PLA/GO/Q suspensions, including density, surface tension, electrical conductivity and viscosity, are presented in Table 1. These physical properties are the main parameters that affect electrospinning applications. Solutions with high electrical conductivity are known to have a downsizing effect on the diameters of fibers. With the addition of different amounts of GO into the PLA, it was clearly observed that the electrical conductivity and surface tension values increased with GO addition. However, the viscosity values of the solutions decreased. With the addition of the Q into the PLA/GO blends, the electrical conductivity values of the solutions increased sharply, and the surface tension values of the solutions decreased with the addition of Q. All of these behaviors are summarized in Table 1 with values.

### 3.2. Morphological Examinations

**Figure 2:**
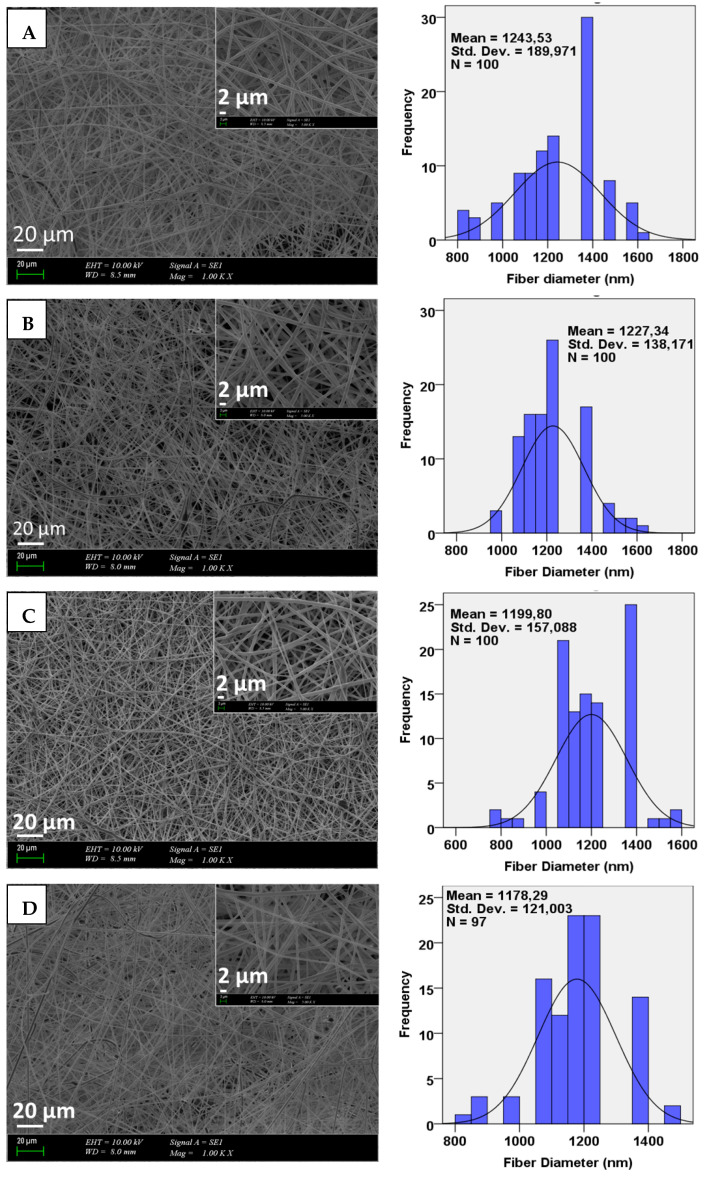

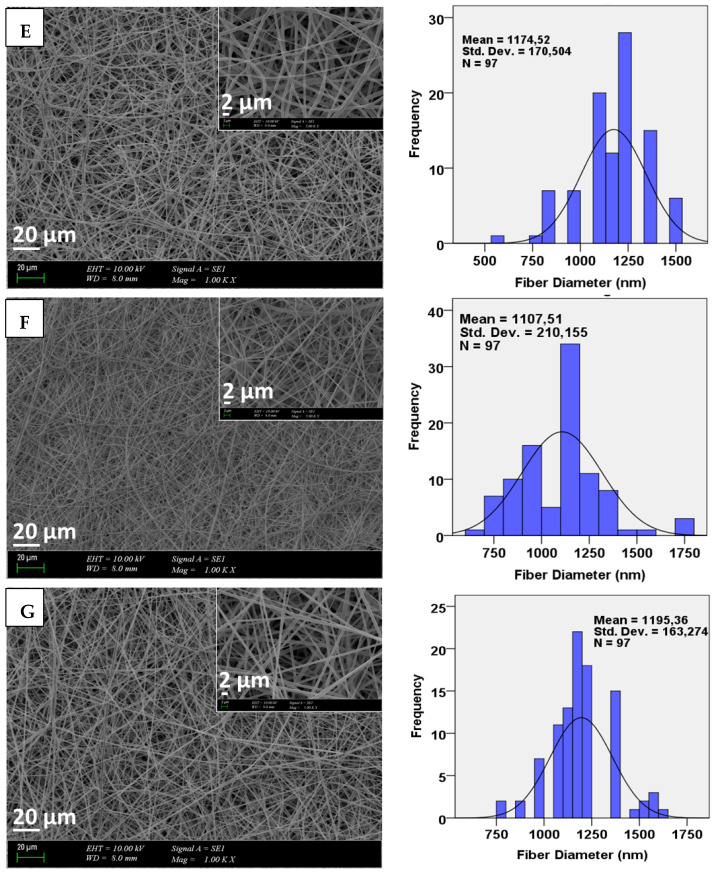
SEM images of the 10%PLA (**A**), 10%PLA/0.1%GO (**B**), 10%PLA/0.5%GO (**C**), 10%PLA/1% GO (**D**) 10%PLA/0.1%GO/Q (**E**), 10%PLA/0.5%GO/Q (**F**), 10%PLA/1%GO/Q (**G**) and their diameter distributions.

The scanning electron microscopy (SEM) results for PLA/GO and PLA/GO/Q microfibers are shown in Figure 2. All microfibers show uniform and smooth surface morphologies without any beads. Figure 2A presents the 10 wt.% PLA microfibers with a 1.24 ± 53 µm histogram graph. By adding 0.1%GO, 0.5%GO, 1% GO into the 10 wt.% PLA matrices, the diameters of the microfibers decreased to 1.22 ± 138 µm, 1.19 ± 157 µm, and 1.17 ± 121 µm, respectively, with no beaded structures, indicating that the selection of electrospinning parameters was correct. In addition, it was proved that the PLA/GO blends are easy to electrospin [35]. Figure 2 also shows the Q-loaded microfiber scaffolds with their diameters. No significant difference was observed in surface morphology, but it was seen that the diameter values of the microfibers decreased with the addition of Q into the PLA/GO blends. The diameters of the Q-loaded scaffolds were 1.17 ± 170 µm, 1.10 ± 210 µm, and 1.19 ± 163 µm for 10%PLA/0.1%GO/Q, 10%PLA/0.5%GO/Q, and 10%PLA/1%GO/Q, respectively. Generally, it can be said that the diameter of the Q-loaded microfibers is smaller than the diameter of the reference microfibers. These results clearly demonstrate that the increase in GO concentration in both the unloaded and Q-loaded microfibers did not cause important changes in morphology, but led to a reduction in the diameter of the fibers.

### 3.3. Fourier Transform Infrared Spectroscopy (FTIR)

**Figure 3:**
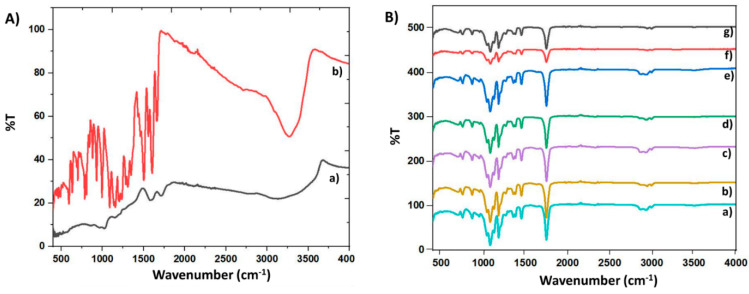

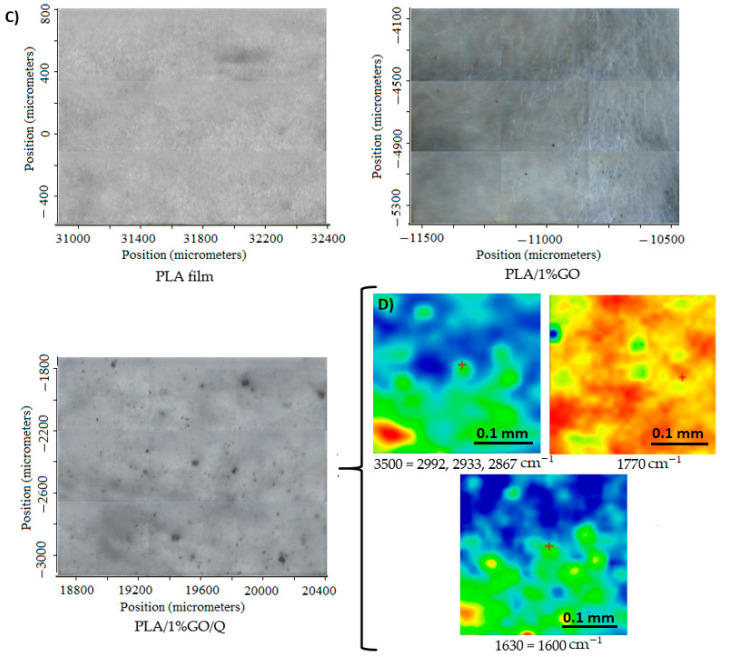
FTIR spectrums of the pristine Q (**A**,a), GO (**A**,b), and their blends: 10%PLA (**B**,a), 10%PLA/0.1%GO (**B**,b), 10%PLA/0.5%GO (**B**,c), 10%PLA/1% GO (**B**,d), 10%PLA/0.1%GO/Q (**B**,e), 10%PLA/0.5%GO/Q (**B**,f), 10%PLA/1%GO/Q (**B**,g). (**C**) Video images recorded on the three samples PLA, PLA/1% GO and PLA/1%GO/Q; and (**D**) FTIR maps recorded on PLA/1%GO/Q at 3500, 1770 and 1630 cm^−1^.

FTIR analysis was performed to determine the chemical groups of the Q, GO, and PLA/GO/Q fibers. The FTIR spectrum of neat Q, synthesized GO, 10 wt.% PLA/(0.1, 0.5, 1) wt.% GO microfibers, and Q-loaded 10%PLA/(0.1, 0.5, 1)% GO microfibers are shown in Figure 3A,B. The main absorption peaks observed at ~3270 cm^−1^ (H-O vibration), ~1600 cm^−1^ (C=C), ~1350 cm^−1^ (C-OH), and ~1280 cm^−1^ (C-O-C) for neat Q are displayed in Figure 3A,a [36,37]. Figure 3A,b presents the synthesized graphene oxide FTIR spectrum, in which four main peaks can be observed. Figure 3A,b shows that GO has a peak at ~1081 cm^−1^, corresponding to the C-O bond, demonstrating the presence of oxide functional groups after oxidation. The peak at ~1630 cm^−1^ indicates that the C = C bond still remains before and after the oxidation process. Water absorbed in GO is indicated by a wide peak from ~2885 cm^−1^ to ~3715 cm^−1^, contributed by O-H stretching of associated H_2_O molecules [38]. Therefore, this peak shows the good adsorption of water onto graphene oxide [39]. Figure 3B,a shows the FTIR spectrum of the PLA microfiber. The main absorption peaks have been reported to be at ~1770 cm^−1^ (C=O vibration), ~1450 cm^−1^ (CH_3_ asymmetrical scissoring), ~1080 cm^−1^ (C-O-C stretching), ~1040 cm^−1^ (C–CH_3_ stretching), and ~860 cm^−1^ (C–COO stretching) [40]. Some shifts were detected with 0.1, 0.5, and 1 wt.% GO addition, proving that GO addition has an important impact because of the strong interactions developing between GO and PLA (Figure 3B,b,c,d). By adding 0.1 wt.% Q into the PLA/GO blends, the peak that is characteristic to Q can be observed at~1350 cm^−1^, which demonstrates the interaction of Q with PLA/GO blends. (Figure 3B,e). Also, the same characteristic peaks can be observed for the spectra of 10%PLA/0.5%GO/Q and 10%PLA/1%GO/Q microfiber scaffolds.

### 3.4. FTIR Microscopy

On the basis of the FTIR spectra, it is very difficult to assess the presence of Q and even the presence of GO in the obtained films, but it is expected that FTIR microscopy could be suitable for identifying heterogeneities. For this purpose, assuming that the highest GO content should lead to the highest agglomeration, and that thus more important heterogeneities will appear, the series involving 1% GO is considered and presented below.

Based on the video images (Figure 3C), important changes can be seen resulting from the presence of additional components in the PLA. All samples highlight the fibrillar structure of the membranes. The addition of GO induces a moderate morphological change due to the appearance of some black areas, presumably GO-based agglomerates. However, it is worth mentioning that when quercetin is added only at a content of 0.1%, the morphology is strongly modified because of the intensification of the dark areas. The explanation for this is, most probably, related to the absorption of the hydrophobic quercetin on the GO, and the agglomeration of this complex into the final membrane.

Plotting the FTIR images according to the desired wavenumbers—3500 (associated OH), 1770 (main PLA band shifted to higher wavelength in composites), 1600 (Q band) and ~1630 cm^−1^ (GO band)—the images presented in Figure 3 were obtained. Based on the spectrum obtained in Figure 3B,g, two peaks that are characteristic of GO and Q can be observed and, based on their similar distribution in the film, they can support the association of these minor components. The FTIR image recorded at 1770 cm^−1^ (the main peak of PLA) is independent compared to the other FTIR images recorded at the peaks characteristic of GO or Q, which are practically similarly distributed, as Q has a good affinity to GO.

### 3.5. Thermal Behaviors of the Scaffolds

**Figure 4:**
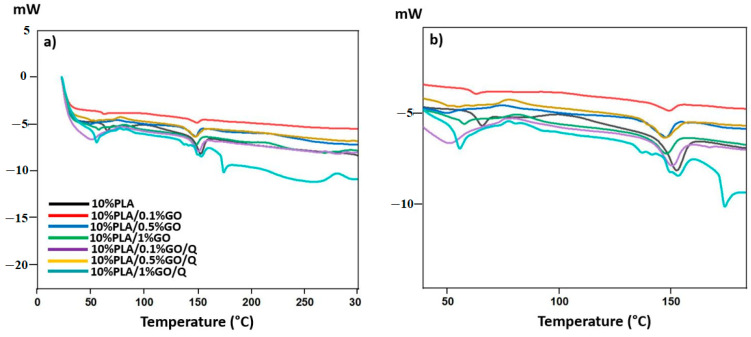
DSC thermograph of all the microfiber scaffolds (**a**,**b**).

Figure 4a,b presents the differential scanning calorimetry (DSC) curves of the fabricated fiber scaffolds. It is known that PLA can have semi-crystalline or amorphous structures, and its glass transition temperature is nearly ~55 °C, while its melting temperature is ~180 °C [41]. On the other hand, these values can change depending on the structures of PLA. In this work, the Tg of PLA was found to be 65.23 °C, a value that is close to the Tg of typical PLA (55 °C) [42]. The melting temperature of PLA was found to be 152.64 °C, which is a value similar to that reported in the study by Kamthai and Magaraphan [43]. Through the addition of 0.1%GO into the 10%PLA, the glass transition and melting temperatures of the PLA changed slightly. However, with the addition of 0.5%GO, the glass transition temperature decreased to 58 °C and the value of the melting temperature decreased to 151.71 °C. With the addition of the highest concentration of GO, the glass transition (61.16 °C) and melting temperature (151.75 °C) of PLA were increased again. With the addition of Q, the glass transition and melting temperatures dropped again. However, an increase was observed for samples with 10%PLA/0.5%GO/Q and 10%PLA/1%GO/Q. Therefore, it can be concluded that the existence of GO in higher amounts induces a decrease in the transition temperatures of the PLA matrix. Moreover, the addition of Q also decreased these values when added into the PLA/GO blends.

### 3.6. Tensile Behavior of the Scaffolds

The stress–strain curves of the PLA, PLA/GO, and PLA/GO/Q microfibers are shown in Table 2. The tensile strength value of PLA was found to be 1.037 MPa. With the addition of 0.1%GO and 0.5%GO into the 10%PLA, the tensile strength value increased to 1.418 ± 0.204 MPa and 1.661 ± 1.469 MPa, respectively. These results are in agreement with the literature data. For example, Liu et al. [44] demonstrated that with the addition of GO into the PLA/15% nanohydroxyapatite nanofibers, the tensile strength increased dramatically (28.4% increment). However, a decrease was observed with the addition of 1% GO into the 10%PLA, probably because of the agglomerations that appear (especially visible in the FTIR microscopy images). The addition of Q into the PLA/GO blends induced an increase in tensile strength values. The highest tensile strength values were observed for the microfiber scaffolds loaded with 0.5%GO (10%PLA/0.5%GO and 10%PLA/0.5%GO/Q). Consequently, 0.5%GO is the most appropriate GO ratio to use in order to increase mechanical strength of the scaffolds. This can be explained by the fact that the 0.5%GO amount can carry the load during the test due to the efficient stress-transfer effect of the 10%PLA matrix, which takes place without developing important agglomerates that can represent defects [45]. When comparing the strain values of the structures, it can be said that the 10%PLA scaffold has the lowest elongation value (7.56%). In addition, with the addition of GO, the elongation amounts increased proportionally, and the highest elongation value (63.37%) was found for the microfiber scaffold containing 1% GO. On the other hand, with the addition of Q into the PLA/GO blends, the elongation value decreased for the 10%PLA/1% GO (43.73%) microfiber scaffold, and the elongation values continued to increase with the addition of Q for other GO ratios. It is important to mention that with the increase in GO content, Q can be adsorbed onto the GO and assist in the dispersion. This explains why the addition of the same amount of Q in the 10%PLA/0.5%GO led to a small increase of ~100 MPa (tensile strength value being 1.469 ± 0.301 Mpa) but, in 10%PLA/1% GO, where the agglomeration tendency is much higher, the presence of Q significantly improved the mechanical properties, from 1.032 ± 0.134 to 1.422 ± 0.089 MPa (~40%) for 10%PLA/1% GO and 10%PLA/1%GO/Q, respectively.

### 3.7. Swelling Behavior

The swelling behavior was studied in order to evaluate the stability of the scaffolds for wound dressing applications. Based on the literature data, PLA has a low water absorption capacity, due to the large number of hydrophobic groups in the chemical structure, which develop strong intermolecular hydrogen bonds between the PLA molecules. Consequently, their availability to form hydrogen bonds with water ensures that the detachment of the PLA molecules rarely takes place and occurs very slowly. However, the addition of GO, which contains a large number of oxygen functional groups, leads to increased polar groups [46,47,48]. These will interact with PLA molecules, and thus the structure will be altered, with more oxygen-rich sites becoming available for water absorption. The increase of GO content in the scaffolds leads to an increase in the water uptake of the blends from 0.35 for the pure PLA to 0.53 for 10%PLA/0.1%GO to 7.06% for 10%PLA/1%GO (being the highest swelling rate) in the first 24 h (Figure 4). Moreover, all formulations exhibited higher swelling capacities than that of pure PLA; while the impact of the addition of 0.1%GO was not important, the addition of 0.5 and especially 1% of GO led to important increases.

The addition of Q into the formulations led to a slight decrease in the swelling rate due to the hydrophobic nature of the Q. Nevertheless, the development of microstructured delivery systems has improved the solubility and stability, while minimizing the degradation process, of these kinds of substances [19,20]. Thus, the samples loaded with Q showed good swelling capacity, with maximum values of 5.17% for 10%PLA/0.5%GO/Q, 4.23% for 10%PLA/0.1%GO/Q and 4.47% for 10%PLA/1%GO/Q in the first 24 h. The swelling behavior was probably influenced by the pore structure of the scaffolds. The addition of Q led to a decrease in the porosity of the scaffolds and in water uptake.

**Figure 5:**
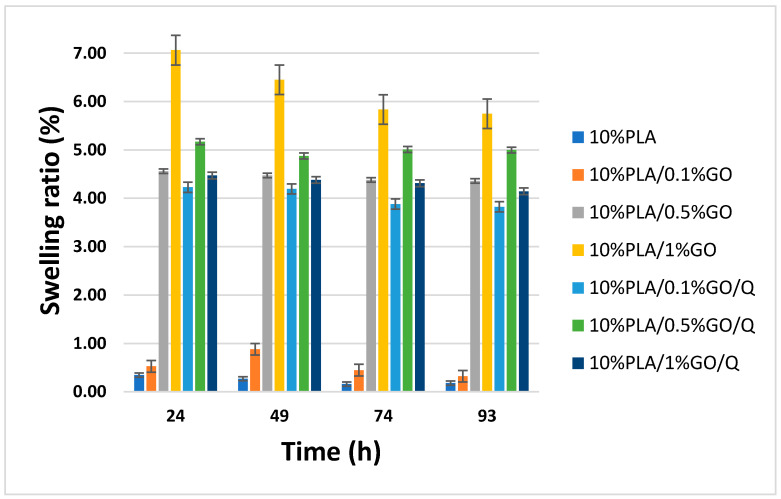
Swelling kinetics of the scaffolds.

After 24 h, a decrease in the swelling of the scaffolds occurred. As shown in Figure 5, the 10%PLA/1%GO displayed higher swelling after 1 day of soaking in PBS (7.06%) than the other scaffolds, followed by a slow decrease, reaching 6.45% on day 2, 5.84% on day 3, and 5.75% on day 4. This could be due to its maximum swelling ratio and large porous structure [49], which allow improved solubilization due to the improved contact of the PLA with the water.

On the basis of their low water absorption, these membranes can be especially recommended for non-suppurated wounds or for outer layers over an adsorbent active wound dressing.

### 3.8. Water Vapor Permeation (WVP)

**Figure 6:**
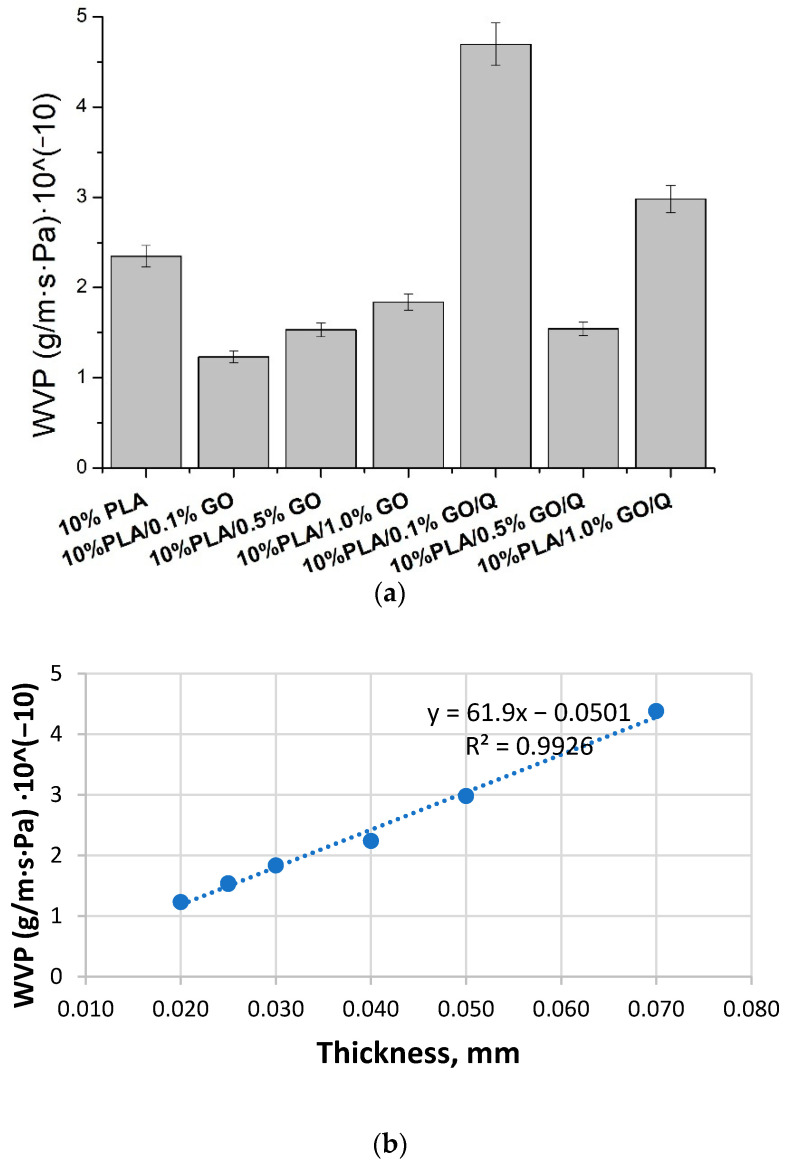
WVP of the PLA membranes (**a**) and WVP versus thickness plotting (**b**).

As can be seen in Figure 6, the WVP of PLA-based membranes is clearly dependent on the thickness of the samples, rather than the composition. For all membranes, the WVP decreases with sample thickness. Table 3 presents the WVP of the samples correlated with the average thickness. By adding GO, the permeability of the membranes increased by ~8% compared to the PLA film. This could be due to the good dispersibility of GO in the PLA matrix, making the membranes more hydrophilic [50,51,52,53]. Liu et al. [44] also demonstrated that the surface wettability of electrospun composite fiber mats is enhanced by the GO and/or nanohydroxyapatite inclusions. The increased hydrophilicity of PLA/PBC/GO fiber membranes was also demonstrated by Gu et al. [48]. To check the dispersibility of GO material in the PLA matrix, the WVP versus thickness is plotted in Figure 6. From this figure, it is obvious that WVP is linearly dependent on thickness. This means that this characteristic is very little influenced by other parameters, with the correlation factor (R^2^) with the equation WVP = f (thickness) being 0.9978. Thus, it can be concluded that WVP is dependent on the content of GO (as long as the GO content is 0.1, 0.5, or 1%), and that no crossing defects are present, allowing a facilitated transfer of the water vapors. These values confirm that the membranes are uniform, and no transversal pores are present, even if, based on the FTIR images, some agglomerates seem to be present. These agglomerates, however, do not affect the barrier functionality of these membranes.

### 3.9. Drug Release Behaviour of the Q under and without Electric Field

The Release Behavior of the Q without Electric Field

**Figure 7:**
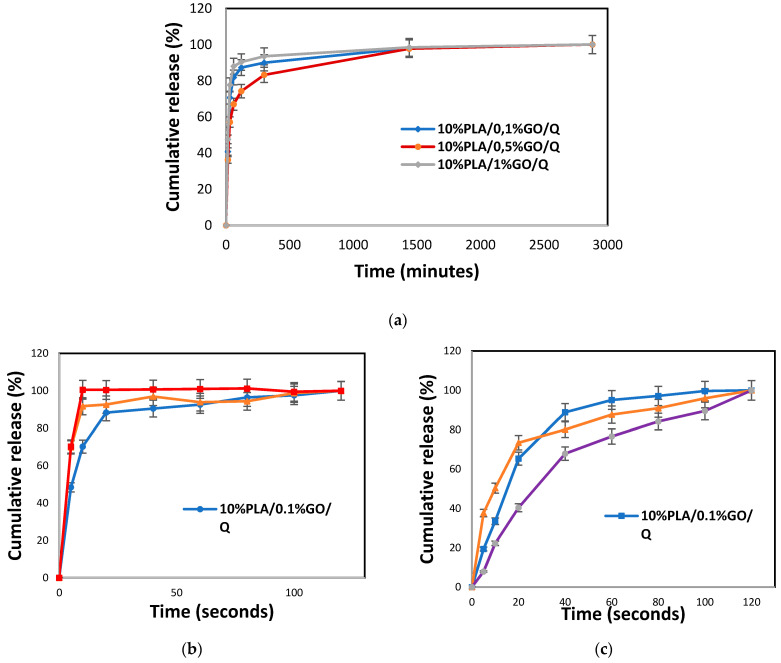
The cumulative release graphs of the drug-loaded microfiber scaffolds without electric stimulus (**a**), and under electric stimulus with two different frequency values: 10 Hz (**b**), 50 Hz (**c**).3.9.2. Electrically Controlled Q Release from the Scaffolds.

The release of Q from the microfiber scaffolds was tested in a phosphate buffer saline solution. The calibration curve of the Q at different concentrations (0.25 µg/mL, 0.5 µg/mL, 1 µg/mL and 1.5 µg/mL), the absorbance graph obtained at 376 nm, and the encapsulation efficiency of the scaffolds are shown in the Supplementary Materials (Figure S2A–C). Figure 7a presents the cumulative release of Q from the microfiber scaffolds based on 0.1, 0.5 and 1.0% GO and Q. The graph indicates that approximately 83.25, 89.96, and 93.48% of Q was released from 10%PLA/0.1%GO, 10%PLA/0.5%GO, and 10%PLA/1%GO, respectively, after 300 min of immersion. The Q release kinetics reached a plateau after 24 h of immersion, and a maximum of 98.05, 97.70, and 98.43% of the drug was released from 10%PLA/0.1%GO, 10%PLA/0.5%GO, and 10%PLA/1%GO, respectively. Since the microfiber diameters of all scaffolds were similar to one another, the drug release behavior was also similar, with the differences being induced by the structuration of the PLA/GO composite fibers and the affinity of the Q to these supports. Thus, based on these results, it can be concluded that Q release can be tuned according to the GO content [54]. Moreover, the encapsulation efficiency of Q into the various Q-loaded PLA/GO blends was calculated. The highest encapsulation efficiency (EE) was obtained for the 10%PLA/1%GO/Q scaffold, with 89 ± 2.56%, and the lowest EE was observed for the 10%PLA/0.5%GO/Q scaffold, with 78 ± 3.2% (Figure S2C—Supplementary Materials).

Triggered delivery is an important approach in drug delivery, and the literature has started to exploit the potential of using internal or external triggering factors to optimize/enhance the delivery rate. In our case, the use of GO was able to confer electrically triggered delivery, and thus we evaluated the influence of the applied electric field. The influence of the electric field on Q release from the PLA/GO microfiber scaffolds was evaluated at two frequencies, as presented in Figure 7b,c. When the release behavior under the electric field with a frequency of 10 Hz was examined (Figure 7b), the highest release percentage after 5 s of stimulation was obtained for the 10%PLA/1%GO/Q microfiber scaffold, with a cumulative release value of 70.25%.

After 10 s of electric stimulation at 10 Hz, practically all of the Q was released from the 10%PLA/1%GO/Q microfiber scaffold. The lowest percentage of Q release was observed in the 10%PLA/0.1%GO/Q microfiber scaffold (~50% at 5 s), and gradually increased until 120 s. In the first 60 s of release, the scaffold with intermediate content of GO exhibited an intermediate delivery rate. At 120 s of electric stimulation, all the obtained PLA/GO/Q scaffolds reached 100% cumulative release.

The electric triggering capacity was also evaluated at the same current intensity and voltage, but at a frequency of 50 Hz. After 5 s of release at 50 Hz, the highest Q release (37.72%) was obtained for the 10%PLA/0.5%GO/Q microfiber scaffold and the lowest release (only 7.89%) was obtained for the 10%PLA/1%GO/Q microfiber scaffold (Figure 7c). This tendency was maintained for 10 and 20 s, but from 40 to 120 s, the fastest delivery was obtained for 10%PLA/0.1%GO/Q and the slowest for 10%PLA/1%GO/Q. The highest release rates were achieved after 120 s, at 100, 100, and 99.99%, which were recorded for the 10%PLA/0.1%GO/Q, 10%PLA/0.5%GO/Q, and 10%PLA/1%GO/Q scaffolds, respectively. At 120 s, the cumulative release was similar for all three scaffolds. The cumulative releases of the scaffolds at 60 s were proportional to the content of GO, and were found to be 95.11, 87.69 and 76.61% for 10%PLA/0.1%GO/Q, 10%PLA/0.5%GO/Q and 10%PLA/1.0%GO/Q, respectively.

Based on the literature data, the delivery rate has to be controlled in order to provide an appropriate release rate and, consequently, the appropriate biological activity, and also because some biological active agents can lose their activity if not released and absorbed in due time by the body [55].

Figure 7b shows that Q was released much faster from the scaffold with the highest amount of GO at 10 Hz (10%PLA/1%GO) compared to 10%PLA/1%GO at 50 Hz. These results can be attributed to changes in the affinity of the Q, but also to changes in morphology occurring as a consequence of the addition of GO.

After comparing the release data of Q from PLA/GO scaffolds, without and under the stimulation of an electric field, it was demonstrated that ~90% and ~100% of the drug was released at 10 Hz, which is 6000 times (10%PLA/0.5%GO/Q) and 8640 times faster (10%PLA/1%GO/Q) than in the case of traditional drug-release method without any stimulation. When applying an electric field of 50 Hz, the data reveal that ~90% and ~100% of Q was released 750 times (10%PLA/0.5%GO/Q) and 864 times faster (10%PLA/0.1%GO/Q) than in the case of traditional drug-release methods.

These results indicate that PLA/GO scaffolds can be used as smart drug delivery systems with electric triggering capacity, and the delivery can be tuned according to the needs of the patient by changing the applied current characteristics. In this case, the delivery can be easily tuned through the use of an electric field, whereby, by changing the frequency, fine tuning can be carried out. Certainly, further optimizations have to be done by changing the electric field characteristics, but it has been demonstrated that these scaffolds can be used as smart scaffolds with capacity to be triggered electrically, meaning that they can find use in personalized medicine.

### 3.10. Microbiological Assessments

Recently, considerable interest has been devoted to the development of biologically active antibacterial and biocompatible scaffolds that are similar analogues of the extracellular matrix (ECM). This type of scaffold must act as a temporary matrix for cell proliferation and ECM deposition, with subsequent ingrowth until the tissues are totally restored or regenerated. The main challenge is to create scaffolds that promote tissue–cell interactions such as adhesion and proliferation, while simultaneously inhibiting bacterial colonization [56].

Samples 10%PLA, 10%PLA/0.1%GO, 10%PLA/0.5%GO, 10%PLA/1%GO, 10%PLA/0.1%GO/Q, 10%PLA/0.5%GO/Q, 10%PLA/1%GO/Q were analyzed for their ability to inhibit microbial growth by assessing logarithmic reduction, population reduction and recovery rate.

**Figure 8:**
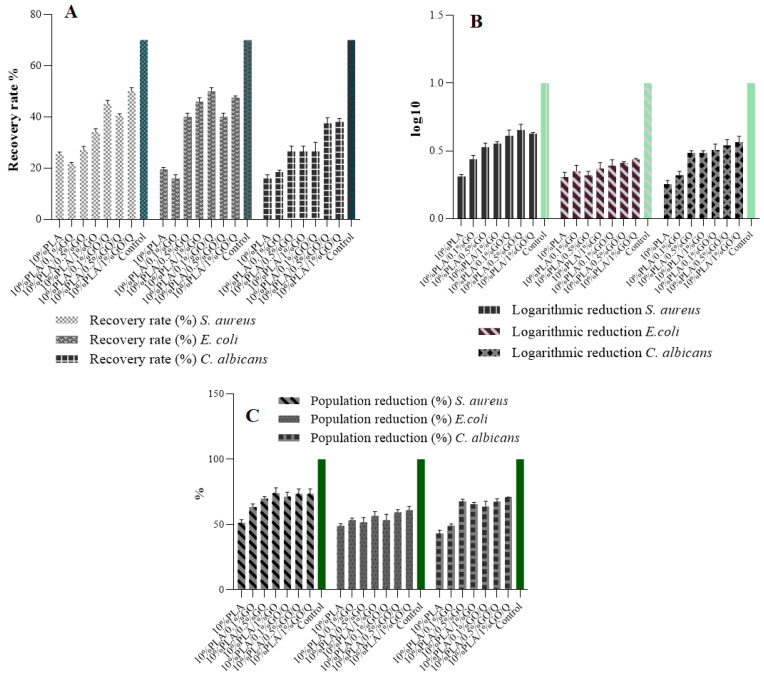
Logarithmic reduction (**A**), population reduction (**B**), and recovery rate (**C**) of *S. aureus, E. coli* and *C. albicans* by 10%PLA, 10%PLA/0.1%GO, 10%PLA/0.5%GO, 10%PLA/1%GO, 10%PLA/0.1%GO/Q, 10%PLA/0.5%GO/Q, 10%PLA/1%GO/Q samples.

The results for logarithmic reduction presented in Figure 8A show a reduction ranging from 0.30 to 0.62 log for the *S. aureus* strain, from 0.28 to 0.43 log for *E. coli* and from 0.23 to 0.53 for *C. albicans*. Logarithm 1 was used as a reference control, representing a 90% microbial reduction. The obtained results indicate modest antimicrobial activity, with reduction rates being similar across all tested strains.

In correlation with the logarithmic reduction, the population reduction (%) results for all tested strains are presented in Figure 8B. A 100% reduction was considered as control for maximum inhibitory effect. For all strains, the reduction percentages ranged between 41% and 76%, indicating a bacteriostatic-like effect in which microbial growth is not promoted.

Recovery rate (%) serves as a very effective screening method when developing or evaluating new materials. When assessing the antimicrobial activity of new materials (with potential use as medical devices), this assay is critical for the accurate determination of antimicrobial effect, disinfection efficacy, bioburden, sterility, or any test that requires the determination of surviving microorganisms in a product possessing antimicrobial properties [57].

The European Pharmacopoeia recommends that, for antimicrobial substances, the recovery rate should not differ by more than a factor of 2 (50–200% recovery). None of the tested samples fits within this interval. Although the tested samples are modest antimicrobials, the bacterial growth is inhibited enough to comply with the specific applications envisioned for these scaffolds.

All in all, it should be kept in mind that fiber diameter [58], surface hydrophilicity, roughness [59], and stiffness [60] are known to affect the ability of bacteria to attach and to proliferate within fibrous networks.

Due to several properties of these membranes, namely stability, suitable WVP despite limited water adsorption capacity, and bacteriostatic capacity, these materials could be recommended as an outer/protective layer that can be applied on an active wound dressing, and which is able to adsorb exudate or can be directly applied on non-suppurated wounds, where liquid absorption is not important.

### 3.11. Biocompatibility Properties of the Scaffolds

The L929 fibroblast cell line was used as a model cell line to evaluate the biological effect of the 10%PLA samples. The MTT tetrazolium salt viability assay was performed to measure the percentage of cell viability after different time intervals, while cell morphology with respect to cell density was evaluated through fluorescence microscopy and SEM.

**Figure 9:**
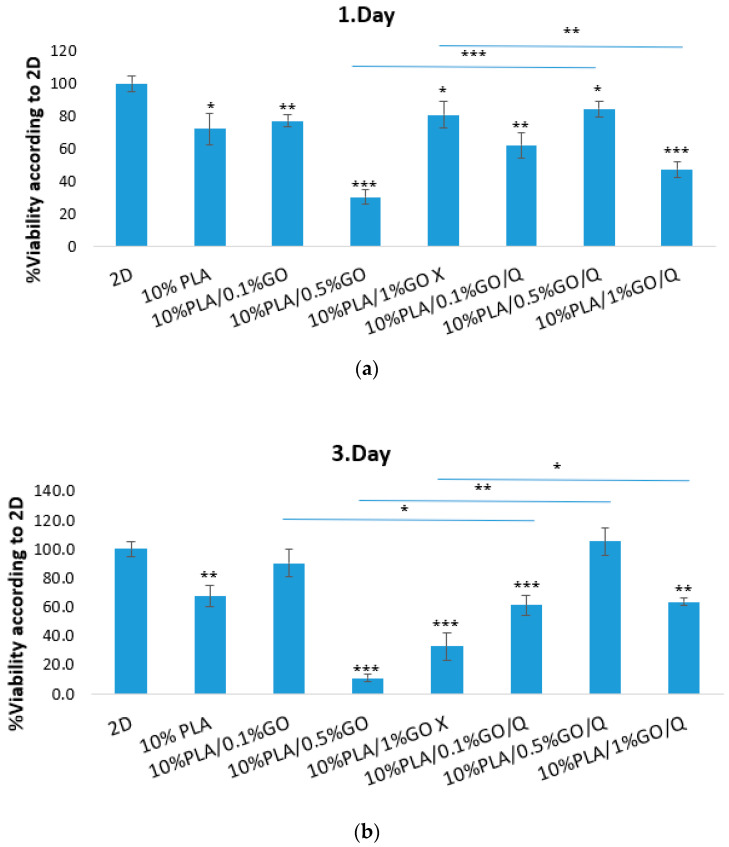

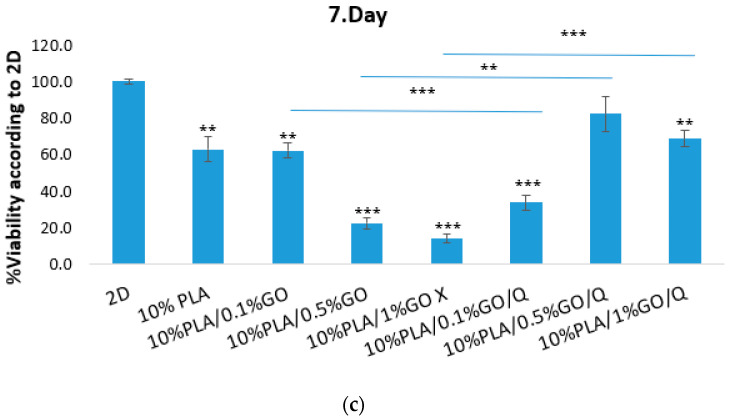
MTT viability assay for L929 fibroblast cells cultivated on the 10%PLA scaffolds at different time intervals; 1 day (**a**), 3 days (**b**) and 7 days (**c**), respectively; data is presented as ±SD. The mark “*” indicates significant difference at *p* < 0.05, “**” *p* < 0.01 and “***” *p* < 0.001.

According to the MTT test for the first-day incubation period, the viability values of all the samples were found to be lower than that of the control group (*p* < 0.05, Figure 9). The lowest viability value (30.1%) belonged to the 10%PLA/0.5%GO microfiber scaffold (*p* < 0.001 compared to controls). However, with the addition of Q to the 10%PLA/0.5%GO microfiber scaffold, the viability value was 84.3% for the first day (*p* < 0.05 with respect to control, and *p* < 0.001 for 10%PLA/0.5%GO vs. 10%PLA/0.5%GO/Q). In contrast, the apparent cell viability of the 10%PLA/1%GO was 80.7% (*p* < 0.05), while for the quercetin-loaded 10%PLA/0.5%GO, a viability value of 47.1% was obtained (*p* < 0.01 compared to control, and *p* < 0.01 for 10%PLA/1%GO vs. 10%PLA/1%GO/Q). The viability of the 10%PLA microfiber scaffold in the first day had a value of 72% (*p* < 0.05). After the third day of incubation, the viability values for 10%PLA (NS), 10%PLA/0.5%GO (*p* < 0.01), 10%PLA/1%GO (*p* < 0.01), and 10%PLA/0.1%GO/Q (NS) microfiber scaffolds decreased compared to the first day of incubation. However, the viability values of the cells cultured with 10%PLA/0.1%GO (NS), 10%PLA/0.5%GO/Q (*p* < 0.05) and 10%PLA/1%GO/Q (*p* < 0.01) samples increased compared to the first day. On the seventh day of culture, the viability values for all of the scaffolds except for 10%PLA/1%GO/Q decreased (*p* < 0.05). The highest viability value (82.3%) on the seventh day was obtained for the 10%PLA/0.5%GO/Q microfiber scaffold (NS compared to controls). The results suggest that the addition of Q in the samples was beneficial, except for the 10%PLA/0.1%GO samples, where a lower viability was observed with the addition of Q at all time intervals.

**Figure 10:**
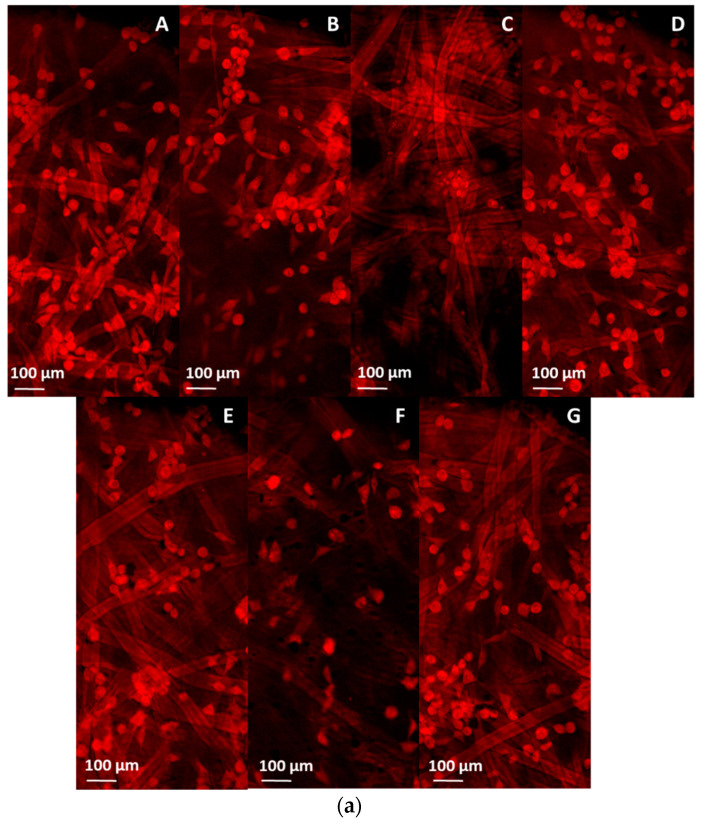

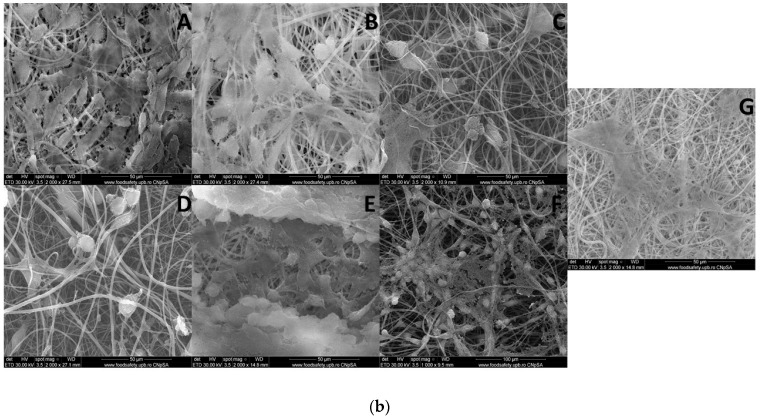
Fluorescence microscopy images (**a**) of L929 fibroblast cells at 24 h after direct interaction with samples and SEM images (**b**) of the fibroblast cells on the microfiber scaffolds: (**A**) 10%PLA (scale bar 50 µm, 2000× magnification), (**B**) 10%PLA/0.1%GO (scale bar 50 µm, 2000× magnification), (**C**) 10%PLA/0.5%GO (scale bar 50 µm, 2000× magnification), (**D**) 10%PLA/1%GO (scale bar 50 µm, 2000× magnification), (**E**) 10%PLA/0.1%GO/Q (scale bar 50 µm, 2000× magnification), (**F**) 10%PLA/0.5%GO/Q (scale bar 100 µm, 1000× magnification), (**G**) 10%PLA/1%GO/Q (scale bar 50 µm, 2000× magnification).

The qualitative morphological observations (fluorescence microscopy and SEM) were in concordance with the MTT data, showing that cells do attach to the electrospun samples, adhering to the microstructured fiber network. Fluorescence images of the fibroblast cells cultured on the microfiber scaffolds were shown in Figure 10a while the SEM micrographs were showcased in Figure 10b. The highest cell density and homogenous distribution of the cells was observed in case of 10%PLA, 10%PLA/0.1%GO and 10%PLA/1%GO/Q samples. An obvious decrease in cell attachment was observed for 10%PLA/0.5%GO. The addition of Q in the samples decreased the cellular density.

The SEM micrographs indicated that the cells have a normal elongated morphology when cultivated on all samples, with the exception of 10%PLA/0.5%GO, 10%PLA/1%GO and 10%PLA/0.5%GO/Q, where the fibroblasts adhered to the fillamentous network of these materials with a round shape.

**Figure 11:**
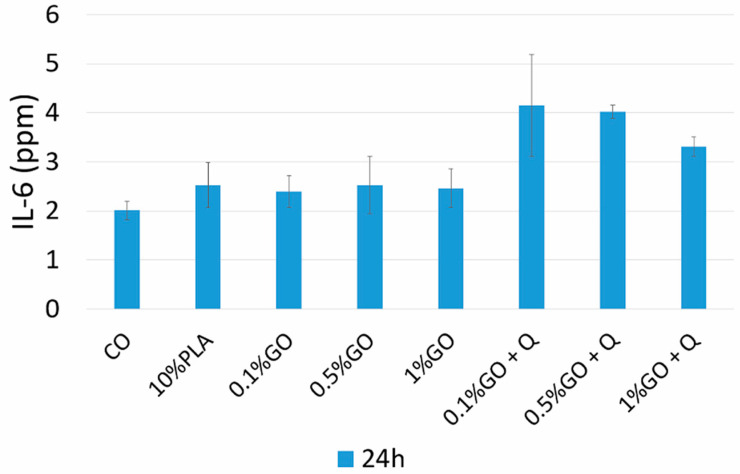
IL-6 release.

Biomaterial extracts are often employed in biocompatibility assessment in order to assess the eventual irritant effect of potential leachates in contact applications, such as wound healing scaffolds [61]. Fibroblast cells are present in the structure of all organs as one of the main components in loose connective tissue, playing an essential role in the inflammation process involved in wound healing [62]. During acute response, fibroblasts can release interleukin 6 (IL-6), and thus the cytokine production was measured here in L929 fibroblast cells after 24 h exposure to biocompatible cell culture medium extracts diluted in a ratio of 1:8 . The results showed that the addition of Q into the samples stimulated the production of IL-6 at 24 h, which could be linked to an acute inflammatory response in the exposed fibroblast cells as a potential effect of wound healing (Figure 11).

By evaluating the biocompatibility test results, it can be said that the effect of cell proliferation depends on the concentration and the duration of cell exposure to the structure [63].

## 4. Conclusions

Novel PLA/GO/Q electrospun scaffolds were prepared using the electrospinning method using three PLA/GO ratios. The swelling behavior indicated an increase of the water uptake with increasing GO content in the scaffolds. The WVP attained for PLA-based membranes was positively correlated with the thickness. By adding GO, the membranes became more hydrophilic, increasing their permeability by ~8% compared to the neat PLA film. The antimicrobial assay showed a reduction rate ranging from 0.30 to 0.62 log for the *S. aureus* strain, from 0.28 to 0.43 log for *E. coli*, and from 0.23 to 0.53 for *C. albicans*.

The coexistence of 10%PLA/0.5 and 1%GO and Q led to good biocompatibility at 1, 3 and 7 days (viability > 80%), being much higher than the biocompatibility of the 10%PLA/0.5%GO scaffold, demonstrating the beneficial effect of Q, especially at higher time intervals, such as 7 days. The addition of Q into the PLA/GO matrix stimulated the production of IL-6 at 24 h, which could be linked to an acute inflammatory response in the exposed fibroblast cells, as a potential effect of wound healing. Electrical stimulation can be applied to the PLA/GO/Q scaffolds in order to increase the process of drug release from the scaffolds. According to these data, the complete release happened in just 1–2 min when an electric field of 10 or 50 Hz was used, while hundreds of minutes were necessary for a similar release without the application of an electric field. Based on these results, it is evident that the addition of GO can induce electric triggering capacity and personalized release. The release and dosage flexibility imparted by the application of an external electric field makes the fibrous scaffold an exciting candidate for on-demand drug delivery with applications in personalized medicine such as wound healing. Supplementary research will be carried out in order to develop new drug delivery systems with electric stimuli triggering capacity using biological active agents with different activities (antitumoral, antimicrobial, etc.). Both material optimization and field characteristics will be considered.

## Figures and Tables

**Table 1:** Physical properties of the microfiber suspensions.

Solutions	Density (g/cm^3^)	Electrical Conductivity (μS/cm)	Surface Tension (mN/m)	Viscosity (mPa·s)
10%PLA	1.31	1.6 ± 0.05	15.9 ± 0.7	5352 ± 213
10%PLA/0.1%GO	1.29	3.7 ± 0.3	16.77 ± 0.2	5802 ± 225
10%PLA/0.5%GO	1.31	6.2 ± 1.3	17.3 ± 0.4	3431 ± 447
10%PLA/1%GO	1.33	9.9 ± 0.3	21.3 ± 1.7	3005 ± 512
10%PLA/0.1%GO/Q	1.31	4.4 ± 0.1	19.6 ± 2.3	3105 ± 237
10%PLA/0.5%GO/Q	1.31	14.2 ± 0.5	15.5 ± 0.5	5085 ± 346
10%PLA/1%GO/Q	1.97	15.6 ± 0.7	15.4 ± 2.6	4672 ± 601

**Table 2:** Mechanical test results obtained from the tensile test.

Microfibers	Tensile Strength (MPa)	Strain at Break (%)
10%PLA	1.037 ± 0.028	7.56 ± 4.193
10%PLA/0.1%GO	1.418 ± 0.204	41.249 ± 5.076
10%PLA/0.5%GO	1.469 ± 0.301	45.475 ± 4.104
10%PLA/1% GO	1.032 ± 0.134	63.369 ± 13.300
10%PLA/0.1%GO/Q	1.143 ± 0.057	54.571 ± 0.474
10%PLA/0.5%GO/Q	1.751 ± 0.819	46.116 ± 7.768
10%PLA/1%GO/Q	1.422 ± 0.089	43.729 ± 24.537

**Table 3:** Water vapor permeation through the membranes for the samples correlated with the average thickness of the membranes.

Microfibers	Average Thickness, (µm)	WVP, (g/m·s·Pa)·10^−10^
10%PLA	40 ± 6	2.35 ± 0.12
10%PLA/0.1%GO	202 ± 1	1.23 ± 0.06
10%PLA/0.5%GO	25 ± 4	1.53 ± 0.08
10%PLA/1%GO	30 ± 5	1.83 ± 0.09
10%PLA/0.1%GO/Q	70 ± 6	4.69 ± 0.24
10%PLA/0.5%GO/Q	25 ± 3	1.54 ± 0.08
10%PLA/1%GO/Q	50 ± 10	2.98 ± 0.15

## Data Availability

Not applicable.

## Supplementary Materials

The following are available online at https://www.mdpi.com/article/10.3390/pharmaceutics13070957/s1, Figure S1: The circuit diagram. Figure S2: The calibration curve of the Q with different amounts (A): 0.25 µg/mL, 0.5 µg/mL, 1 µg/mL and 1.5 µg/mL, absorbance graph obtained at 376 nm (B), encapsulation efficiency of the scaffolds (C).
