# Supplementary material for: Electrically Triggered Drug Delivery from Novel Electrospun Poly(Lactic Acid)/Graphene Oxide/Quercetin Fibrous Scaffolds for Wound Dressing Applications
Source: Pharmaceutics. 2021 Jun 25;13(7):957. doi: 10.3390/pharmaceutics13070957 (PMC8309188; doi:10.3390/pharmaceutics13070957)
Supplement: Supplementary file 1 [file pharmaceutics-13-00957-s001.zip › pharmaceutics-1203388-supplementary.pdf]

pharmaceutics

MDPI

Supplementary Materials: Electrically Triggered Drug Delivery from Novel Electrospun Poly(lactic acid)/Graphene Oxide/Quercetin Fibrous Scaffolds for Wound Dressing Applications

Alexa-Maria Croitoru, Yasin Karaçelebi, Elif Saatcioglu, Eray Altan, Songul Ulag, Huseyin Kıvanc Aydoğan, Ali Sahin, Ludmila Motelica, Ovidiu Oprea, Bianca-Maria Tihauan, Roxana-Cristina Popescu, Diana Savu, Roxana Trusca, Denisa Ficai, Oguzhan Gunduz and Anton Ficai

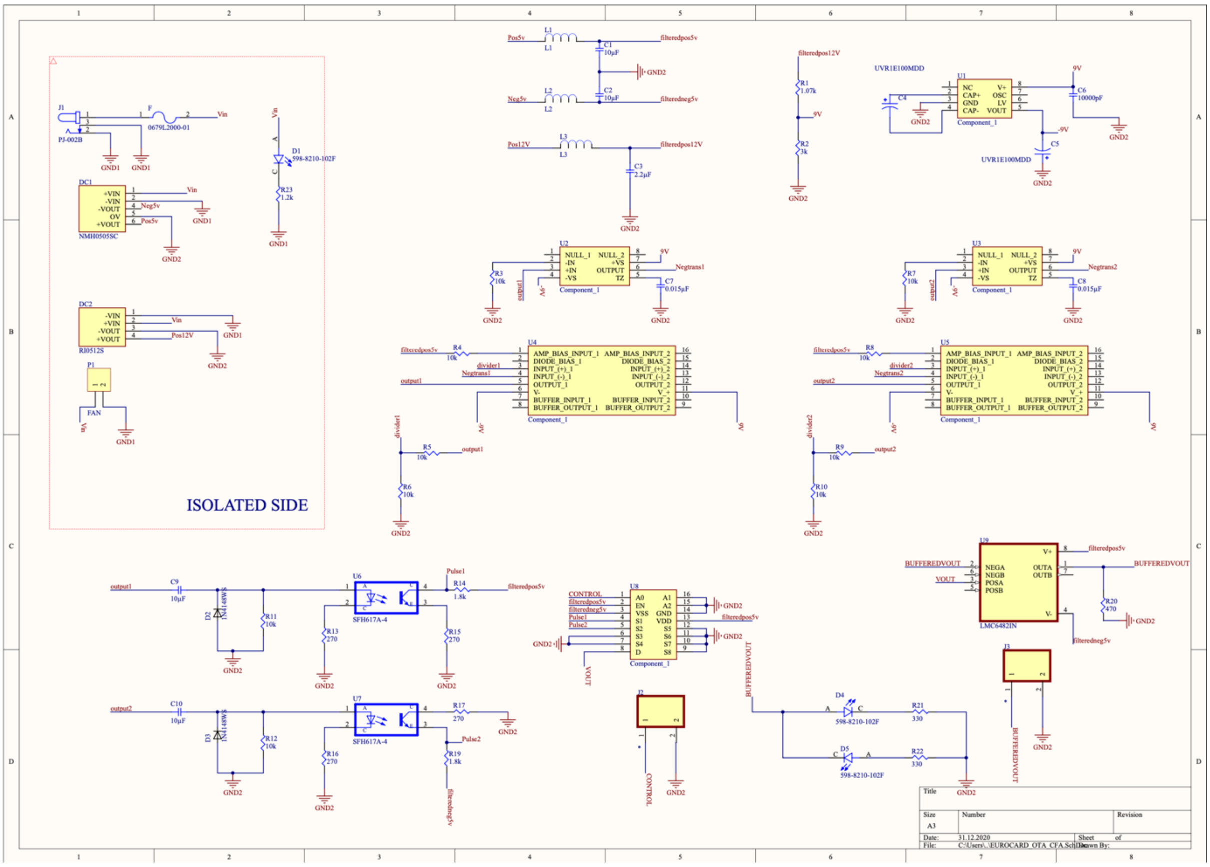

Figure S1. The circuit diagram.

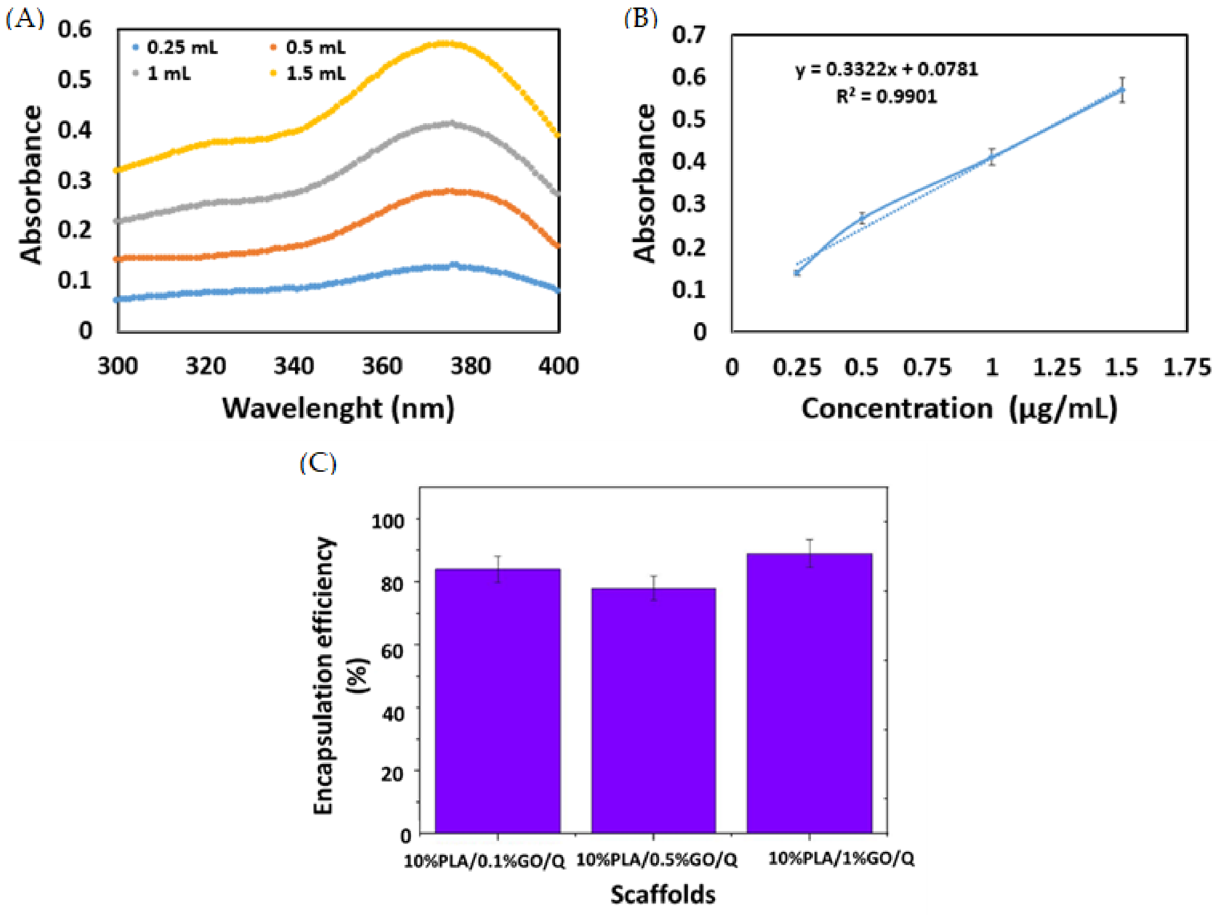

Figure S2. The calibration curve of the Q with different amounts (**A**): 0.25 µg/mL, 0.5 µg/mL, 1 µg/mL and 1.5 µg/mL, absorbance graph obtained at 376 nm (**B**), encapsulation efficiency of the scaffolds (**C**).
